# Resolvins in Periodontitis and Possible Periodontal Regeneration: A Literature Review

**DOI:** 10.7759/cureus.68187

**Published:** 2024-08-30

**Authors:** Sneha Chiluveru, Mrunalini Gundelly, Santosh V Pusuluri, Manasa Tummanepally, Meenakshi Chandaka, Rekha R Koduganti

**Affiliations:** 1 Department of Periodontics, Panineeya Mahavidyalaya Institute of Dental Sciences, Hyderabad, IND

**Keywords:** periodontal tissue regeneration, resolvins, host modulation, oxidative stress, periodontitis

## Abstract

Periodontitis is a rampant global disease with multifactorial etiology. The main harbinger of periodontitis is the plaque biofilm. The mature biofilm in turn interacts with the micro-organisms and the host, with environmental and genetic factors as additional initiators to cause disease. There are several strategies of preventive periodontics which include host modulation therapy to ameliorate the disease. Recently a lot of research has been done related to the role of resolvins in periodontitis. This article showcases the role of resolvins in periodontal health and disease.

## Introduction and background

Periodontitis is a chronic inflammatory disease caused by the formation of a plaque biofilm which, after maturation, results in the loss of alveolar bone and periodontal connective tissue, causing tooth mobility and ultimately tooth loss [1]. Nowadays, surgery and other therapeutic strategies for periodontitis are aimed at controlling local inflammation and removing plaque to minimize symptoms and prevent disease progression [2,3]. However, total regeneration of the lost periodontal tissues is not possible, though regenerative approaches like guided tissue regeneration and bone grafts, when employed, have improved the treatment outcomes [4,5]. Hence other techniques have to be developed that have a good potential for tissue regeneration. An infection or damage disturbs the natural tissue homeostasis, causing acute inflammation which is one of the biological processes that are crucial to the immunological host response. To allow the afflicted tissue to restore to its pre-inflammatory state and function, the inflammatory process is designed to stop. The passive nature of inflammatory resolution was once thought to be caused by a programmed decrease in leukocyte activity due to the inhibition of chemokine signaling.

When Metchnikoff [6] discovered that tissue macrophages ingest neutrophils, and acute inflammation ends, it was a significant discovery. Polymorphonuclear neutrophils (PMNs) are the first leukocyte responders to gather at the inflammatory site; they are followed by monocytes, macrophages, and mononuclear cells. By entering the inflammatory site and aiding in the removal of cellular debris, the macrophages phagocytize apoptotic polymorphonuclear neutrophils, which in turn prolong the inflammatory response.

Nevertheless, if the toxic metabolites of neutrophils and apoptotic inflammatory cells are not eliminated, the lesion becomes chronic, which can result in chronic periodontal disorders [7]. However, data from research conducted in the last few decades indicates that the resolution of inflammation is a deliberately planned, active process [8]. Serhan et al. discovered particular pro-resolving pathways and lipid mediators which created a paradigm shift and provided fresh insight into how inflammation resolves [9]. It is now commonly acknowledged, in contrast to earlier ideas, that it involves a series of overlapping events wherein proinflammatory mediators either activate their receptor targets or signal the creation of specialized pro-resolving mediators (SPM). Put differently, there exists a close relationship between the mediators that trigger acute inflammatory reactions and the signals that control how quickly these responses resolve [10]. Consequently, the peak of the acute inflammatory response is regarded as the start of resolution, as it will be explained in more detail later, indicating where the "Beginning programs the end."

A class of endogenous compounds known as resolvins, lipoxins, protectins, and maresins are examples of specialized immune resolvents that actively promote the reduction of inflammation. This review aims to highlight the newer strategies to treat periodontitis and also emphasizes the role of resolvins in in vitro and in vivo studies to achieve successful treatment outcomes [11].

## Review

Specialized pro-resolving mediators

The SPM known as resolvins (Rv), protectins, and maresins prevent further neutrophil recruitment to the site of inflammation and encourage macrophage clearance of debris, apoptotic cells, and germs [12]. Furthermore, the SPM has strong effects on reducing inflammation and encouraging tissue regeneration and wound healing. These powerful autacoids are now recognized for their capacity to stereo-selectively promote inflammation/resolution by reducing leukocyte responses. They were first discovered using temporal lipidomics with self­ resolving exudates [13]. Specialized pro-resolving lipid mediators are the derivatives of essential fatty acids that can serve as futuristic pharmacological aids for resolving chronic inflammations. They consist of the eicosapentaenoic acid (EPA) derivatives RvE1-RvE3, docosahexaenoic acid (DHA) derivatives RvD1-RvD6, arachidonic acid derivatives which include lipoxin (LX) A4 and LXB4, protectins, maresins, and aspirin-triggered epimeric forms such as omega3 and omega 6 fatty acids.

Structure of Resolvins

Resolvins are lipid mediators derived from omega-3 polyunsaturated fatty acids (PUFA). Based on their precursor fatty acid composition, they are divided into two major categories.

E-series resolvins (RvE):They are formed from 5Z, 8Z, 11Z, 14Z, 17Z-EPA, through cyclooxygenase 2 (COX2) and 5-lipoxygenase (5-LOX) pathways. The combined action of leukocyte and endothelial cells promotes their synthesis. The production of E-series resolvins is also attributed to the cytochrome P450-driven route, which is an aspirin-independent mechanism of EPA. Increased levels of 5-LOX during inflammation trigger an increase in RvE production. On the other hand, the eosinophils produce RvE3 via the 12/15-LOX pathway.

D-series resolvins (RvD)*: *The catalysis of 15-lipoxygenase (15-LOX) and 5-LOX are required for the synthesis of these resolvins. Leukocytes, endothelial cells, neutrophils, and macrophages are examples of cells that offer an appropriate environment for the resolution process. The aspirin-acetylated COX-2 route is another recognized mechanism for RvD synthesis. Lipid oxidation, hydrolysis, and epoxidation are steps in the formation of activated RvDs. The pathway of synthesis of RvD3-RvD6, however, has not yet been documented. Resolvins' structure usually consists of a polyunsaturated fatty acid backbone with certain oxygenation patterns, like conjugated double bonds and hydroxyl groups, which are essential to their biological function.

Generation of resolvins

A class of important lipids that humans cannot synthesize from scratch is the w-3 fatty acid family. The two main long-chain w-3 fatty acids found in food are EPA and DHA. Since algae are the primary producers of these vital fats in the ecosystem, fish, which consume a lot of algae, have high EPA and DHA content. Intake of these nutrients through diet is required because the enzymatic conversion of the parent a-linolenic acid to EPA and DHA in humans appears to be insufficient [14]. Acute inflammatory responses cause rapid fluctuations in the permeability and perfusion of adjacent blood vessels. Not only do these events allow circulating leukocytes and plasma proteins to migrate, but they also offer a way to transport EPA and DHA from the circulation to the site of inflammation. Oedema and the extravasation of serum proteins occur at the site of the inflammatory lesion at the same time as SPM precursors, such as EPA and DHA. This discovery emphasizes the critical function that edema plays in the efficient delivery of w-3 PUFAs to the inflammatory tissue through serum protein exudation [15]. Aspirin plays a triggering role in one well-documented pathway that leads to the production of RvE1. In actuality, acetylation of COX2, a protein found in vascular endothelial cells, can occur when aspirin is administered locally at areas of inflammation. The inflammatory exudate contains EPA, which can be converted to 18R-hydro (peroxy)­ eicosapenataenoic acid (EPE) via the acetylated form of COX2. This is soon reduced to 18R-hydroxyeicosapentaenoic acid (18R-HEPE), which then is acted upon by 5-LOX which quickly converts it to RvE1 [16]. It is also possible to produce resolvin E1 using the microbial P450 route. Furthermore, leukotriene BS (LTBS), which is produced by the host's 5-LOX, can be processed by the same enzyme cytochrome P450 monooxygenase to make RvE1. Interestingly, RvE1 can also be biosynthesised by Candida albicans. In C. albicans, RvE1 production takes place without the assistance of other cellular partners, in contrast to the transcellular biosynthesis of human resolvins [17]. Though human PMNs exhibit increased phagocytosis and hydroxyl-radical­ mediated death of C. albicans in the presence of RvE1, it is unclear how RvE1 synthesis benefits C. albicans.

A systematic review conducted by Nouf Alshibani included five studies that investigated the role of resolvins in experimental periodontitis in animals. New Zealand white rabbits were used in three studies, Wistar rats and Albino mice in two studies, respectively. Four studies evaluated eicosapentaenoic acid-derived RvE1, and one study evaluated docosahexaenoic acid-derived RvD2. Oral-topical application of Rv was followed in four studies, and intra-peritoneal Rv injection was administered in one study. The study duration in these studies ranged between four to 12 weeks, and the Rv dose was between 0.1 μg to 0.5 μg. It was concluded from this review that resolvins have an inhibitory effect on the inflammatory process as well as alveolar bone loss in experimental animal models paving a role for them to be studied in periodontal regeneration [18].

Another human study evaluated the adjunctive treatment of chronic periodontitis with omega-3 fatty acid and low-dose aspirin. Two groups, 40 subjects in each were included. The control group received scaling and root planing and placebo whereas the test group received scaling and root planing along with 900 mg of omega 3 fatty acids and 81 mg of aspirin. The plaque index (PI), gingival index (GI), bleeding on probing (BOP), Probing depth (PD), and clinical attachment loss (CAL) were measured at baseline, three months, and six months. The PD and CAL reduced significantly at three months and six months in the test group compared to the control group. Related to the PI and GI there was no significant difference between the two groups at three months and six months. Related to the biochemical outcome there was a statistically significant reduction in receptor activator of nuclear factor kappa-Β ligand (RANKL) concentration in saliva in the test group at three months and six months. Also, the salivary matrix metalloproteinases (MMP)-8 levels were statistically lower in the test group at six months compared to the control group [19].

Rampally et al. in their study compared the effectiveness of low-dose aspirin versus omega-3 fatty acids as adjuvants to nonsurgical periodontal therapy (NSPT) in Type II diabetic patients with chronic periodontitis. They observed that though the clinical parameters and glycosylated hemoglobin levels improved after NSPT in the test groups, a significant improvement in the serum pentraxin levels was observed only in the group that was administered with omega-3 fatty acids [20]. 

Resolvins and periodontitis

Periodontitis is highlighted as the host response's breakdown of connective tissue and bone. Pattern recognition receptors (PRRs) are used to identify bacteria in the biofilm and specific bacterial components during periodontal inflammation. The term "pathogen­ associated molecular patterns" (PAMPs) refers to the molecules that are extensively shared by microbes and are detected by toll-like receptors that are present in host cells. These molecules include lipopolysaccharides, lipoproteins, and lipoteichoic acids. It has been demonstrated that the recognition of PAMPs through binding to these PRRs causes the production of chemoattractant proteins, or chemokines, which draw PMNs to the infection site [21].

Extravasated PMNs pass through the dentogingival apparatus to enter the crevice after entering the gingival tissues. Their extravasation involves significant morphological alterations and is governed by the joint action of high- and low-affinity sticky contacts [22]. While tissue-derived chemokines cause leukocyte integrins to alter and become high-affinity, tissue-derived cytokines increase the expression of endothelial adhesion molecules [23]. Selectins and their ligands mediate the first contact, which is defined as "tethering" with the walls of the blood vessel and attaching to endothelial cells. Leukocytes express L­ selectin, while activated endothelium cells and platelets produce P- and E-selectins. PMNs in circulation are slowed down by tethering, which also promotes integrin­ mediated interactions that allow leukocytes to stick firmly to endothelial cells. The firm attachment of the PMNs to endothelium is controlled by 2-integrins and lymphocyte function-associated antigen1 through their interactions with endothelial counter­ receptors. The events listed above allow PMNs to squeeze through into the diseased tissue by stopping them from moving along the arterial wall. A "feedback loop" controls the amount of PMNs in the tissues to prevent PMN-induced tissue damage. Upon undergoing apoptosis, recruited PMNs are eliminated by macrophages. It has been reported that PMN phagocytosis of microbial or injured tissue cells triggers their apoptosis, which is a crucial stage in the healing of inflammation.

The etiology of periodontitis is significantly influenced by inflammatory lipid mediators. Particularly, exogenous prostaglandin E2 (PGE2) and leukotriene B4 (LTB4) which are strongly associated with the course of the illness, play a major role in the chronic lesion's promotion [23]. As opposed to this, inflammation resolution is a dynamic process, and homeostasis cannot be achieved until neutrophils have left the lesion. According to recent studies, PGE2 and LTB4 stimulate neutrophil recruitment and are involved in the associated tissue damage [24]. Resolvins, as opposed to nonsteroidal anti-inflammatory medications (NSAIDs) that block COX suppression or receptor antagonists, heighten the resolution of inflammation in a feedforward, receptor agonist-driven way [25]. Resolvins play a role in adaptive immune cell reactions as well. When PMA/ionomycin, stimulated human CD4+ and CD8+ T-cells the RvD2 and RvD1 decreased the production of tumor necrosis alpha and interferon-gamma (IFN-y) [26]. D-series resolvins have also been shown to be crucial for cell differentiation and resolution since they enhance the development of regulatory T-cells while inhibiting the production of activated Th1 and Th17 cells. They bind to the cell surface receptor GPR18/DRV2 providing significant protection in infection-induced inflammation [27].

Mizraji et al. conducted a study in mice and stated that P. gingivalis-induced periodontal bone loss is prevented by D-series resolvins, or RvD2. To preserve bone, RvD2 lowers the receptor activator of nuclear factor kappa B ligand/osteoprotegerin ratio in gingival tissues and suppresses CD4+ T-cells' production of IFN-y. RvD2 treatment increases neutrophils in circulation but significantly reduces neutrophils in tissues. RvD2 has the opposite effect on macrophages; in gingiva, it boosts M2 macrophages and lowers monocytes in the bloodstream. Following RvD2 therapy, gingiva exhibits lower mRNA levels of proinflammatory cytokines, whereas interleukin (IL)-10 levels increase. RvD2 suppresses Th1-type adaptive responses, which are known to be involved in alveolar bone loss, by limiting excessive innate inflammatory responses [28].

By impairing innate immune processes and constitutively priming CD4+ T-cells by DCs, type-1 IFNs contribute significantly to the pathophysiology of periodontal disease. This leads to increased RANKL expression and, ultimately, alveolar bone loss. Blocking type-1 IFN signaling, on the other hand, was observed to reduce alveolar bone loss and the Th 1 response. It is interesting to note that after multiple P. gingivalis challenges, the RvD2 can return IFN-a to homeostatic levels, halting the chronic generation of IFN-y and the advancement of the disease. RvD2 therapy decreases IFN-a expression right after infection, inhibiting dendritic cell (DC) antigen presentation and thus increasing the levels of IL-10. This cytokine is recognized for its ability to negatively regulate IFN-a and impede type-1 IFN levels from reappearing. The exact method by which RvD2 affects IFN-a production is currently unknown. It has been proposed that the expression of RANKL on CD4+ T-cells may directly influence the intricately controlled network of bone homeostasis related to bone loss, and that osteoprotegerin (OPG) is a crucial regulator of the survival, activation, and differentiation of osteoclasts in experimental periodontitis [29].

P. gingivalis has been shown to influence bone loss and regulate the RANKL-OPG axis. Treatment with RvD2 lowered the RANKL/OPG ratio, according to Chiurchiù et al. These findings imply that RvD2 therapy inhibits osteoblast and T-cell-mediated signaling of osteoclast development via RANK thus ameliorating bone loss [30].

Innate immune mechanisms are likely regulated by RvD2 activity at numerous regulatory checkpoints, which are essential for initiating pathogenic T-cell-mediated immunity. RvE1-mediated inflammatory resolution decreases neutrophil infiltration, induces neutrophil apoptosis, and draws non-phlogistic macrophages that phagocytize pathogens and apoptotic neutrophils, thus curbing the lesions to eliminate chronic inflammation. The release of proinflammatory cytokines and chemokines is decreased during the resolution of inflammation.

ChemR23, a protein primarily expressed in the scavenger cells, is bound tightly to the resolvins. Therefore, dendritic cells and macrophage migration are controlled by RvE1. Human macrophages also exhibit improved phagocytosis after being incubated with RVE1. The binding of RvE1 to ChemR23 improves macrophage phagocytosis of apoptotic PMNs, which in turn encourages clearance of the inflammatory lesion, a crucial stage in the resolution of inflammation [31].

Apart from its function via ChemR23, RvE1 also carries out its pro-resolving function by attaching itself to leukotreine B4 receptor 1 (BLT1). BLT1 is a high-affinity receptor and is extensively expressed on PMNs and osteoclasts. LTB4, a pro-inflammatory mediator, is primarily generated by activated leukocytes and promotes PMN, eosinophil, and macrophage activation and chemotaxis. It has been demonstrated that RvE1 competes with LTB4 binding as a partial agonist. Thus, LTB4-mediated leukocyte infiltration and activation are inhibited by RvE1, a crucial counterregulatory mechanism in the resolution of inflammation [32].

Resolvins and possible periodontal regeneration

It is widely acknowledged, following decades of periodontal regeneration research, that normal periodontal function can be restored only if the tissues of the periodontium are regenerated or restored. Since cementum regeneration directly affects the quality of periodontal regeneration, it is regarded as the gold standard [33]. Resolvins modulate the mitogen-activated protein kinase (MAPK) and nuclear factor kappa-light-chain­ enhancer of activated B cells (NF-KB) signaling pathways [34]. This modulation leads to decreased expression of inflammatory mediators and increased expression of factors that promote tissue repair and regeneration. They directly stimulate cementoblasts, the cells responsible for producing cementum. This stimulation increases the deposition of new cementum matrix. They also promote the differentiation of precursor cells into cementoblasts, enhancing the pool of active cells available for cementum regeneration. Resolvins inhibit the differentiation and activity of osteoclasts, the cells responsible for bone resorption. This action helps maintain the structural integrity of the alveolar bone, which supports the cementum. They also enhance the synthesis of collagen and other extracellular matrix components by cementoblasts. It was studied that the cementoblast biomineralization is accelerated by RvD1 and RvE1.

Bozkurt et al. recently conducted a study on immortalized mouse cementoblasts treated with different concentrations of RvD1 and RvE1, which demonstrated that MMP-1, MMP-3, and MMP-9 mRNA expressions were decreased in response to RvD1 and RvE1. Thus, it was reported that RvD1 and RvE1 regulate proliferation, mineralization, and suggested a targeted therapeutic approach for cementum turnover during periodontal regeneration and gene expression in cementoblasts [35].

Gao et al. demonstrated that RvE1 did not alter the expressions of alkaline phosphatase (ALP), bone sialoprotein (BSP), or runt-related transcription factor 2 (RTX2), in the mouse caIvarial osteoblast cell line (MC3T3), though this effect might be cell-specific. They observed that RvE1 has a direct action on the bone which facilitates the differentiation of osteoclasts, and bone remodeling by rescuing OPG production and maintaining a favorable RANKL/OPG ratio [36].

Chen et al. reported that RvE1 increased BSP expression and ALP function in dental pulp stem cells. They can inhibit MMPs, enzymes that degrade extracellular matrix components. This inhibition helps preserve the integrity of the newly formed dentin matrix [37].

## Conclusions

Resolvins derived from omega-3 fatty acids emerge as promising bioactive molecules with significant implications for managing periodontitis and promoting periodontal regeneration. Through their multifaceted actions, including anti­ inflammatory effects, promotion of tissue repair pathways, modulation of immune responses, stimulation of angiogenesis, and influence on matrix remodeling, they play a pivotal role in resolving inflammation and supporting the regeneration of periodontal tissues. The potential therapeutic applications of resolvins in dentistry, particularly in the treatment of periodontal diseases, highlight their ability to not only mitigate inflammation but also enhance the natural healing processes of periodontal tissues. Future research efforts aimed at elucidating specific mechanisms and optimizing delivery methods of resolvins could further enhance their clinical utility in periodontal regeneration therapies.

## References

[1] Preshaw PM, Alba AL, Herrera D, Jepsen S, Konstantinidis A, Makrilakis K, Taylor R (2012). Periodontitis and diabetes: a two-way relationship. Diabetologia.

[2] Deas DE, Moritz AJ, Sagun RS Jr, Gruwell SF, Powell CA (20002016). Scaling and root planing vs. conservative surgery in the treatment of chronic periodontitis. Periodontol.

[3] Smiley CJ, Tracy SL, Abt E (2015). Evidence-based clinical practice guideline on the nonsurgical treatment of chronic periodontitis by means of scaling and root planing with or without adjuncts. J Am Dent Assoc.

[4] Lin Z, Rios HF, Cochran DL (2015). Emerging regenerative approaches for periodontal reconstruction: a systematic review from the AAP Regeneration Workshop. J Periodontol.

[5] Li F, Yu F, Xu X (2017). Evaluation of recombinant human FGF-2 and PDGF-BB in periodontal regeneration: a systematic review and meta-analysis. Sci Rep.

[6] Gordon S (2016). Elie Metchnikoff, the man and the myth. J Innate Immun.

[7] Headland SE, Norling LV (2015). The resolution of inflammation: principles and challenges. Semin Immunol.

[8] Muralidharan R, Basavaraju S, Priyadharshini V (2023). Resolvins: the resolving mediators divulged. Int J Appl Dent Sci.

[9] Serhan CN, Hamberg M, Samuelsson B (1984). Lipoxins: novel series of biologically active compounds formed from arachidonic acid in human leukocytes. Proc Natl Acad Sci U S A.

[10] Serhan CN, Clish CB, Brannon J, Colgan SP, Chiang N, Gronert K (2000). Novel functional sets of lipid-derived mediators with antiinflammatory actions generated from omega-3 fatty acids via cyclooxygenase 2-nonsteroidal antiinflammatory drugs and transcellular processing. J Exp Med.

[11] Balta MG, Loos BG, Nicu EA (2017). Emerging concepts in the resolution of periodontal inflammation: a role for resolvin E1. Front Immunol.

[12] Serhan CN (2004). A search for endogenous mechanisms of anti-inflammation uncovers novel chemical mediators: missing links to resolution. Histochem Cell Biol.

[13] Arterburn LM, Hall EB, Oken H (2006). Distribution, interconversion, and dose response of n-3 fatty acids in humans. Am J Clin Nutr.

[14] Fredman G, Serhan CN (2011). Specialized proresolving mediator targets for RvE1 and RvD1 in peripheral blood and mechanisms of resolution. Biochem J.

[15] Chiang N, Serhan CN (2006). Cell-cell interaction in the transcellular biosynthesis of novel ω-3-derived lipid mediators. Cell-Cell interactions. Methods in Molecular Biology.

[16] Arita M, Clish CB, Serhan CN (2005). The contributions of aspirin and microbial oxygenase to the biosynthesis of anti-inflammatory resolvins: novel oxygenase products from omega-3 polyunsaturated fatty acids. Biochem Biophys Res Commun.

[17] Haas-Stapleton EJ, Lu Y, Hong S (2007). Candida albicans modulates host defense by biosynthesizing the pro-resolving mediator resolvin E1. PLoS One.

[18] Alshibani N (2022). Resolvins as a treatment modality in experimental periodontitis: a systematic review of preclinical studies. Cureus.

[19] El-Sharkawy H, Aboelsaad N, Eliwa M (2010). Adjunctive treatment of chronic periodontitis with daily dietary supplementation with omega-3 fatty acids and low-dose aspirin. J Periodontol.

[20] Rampally P, Koduganti RR, Ganapathi SN, Panthula VR, Surya PJ (2019). Comparison of effectiveness of low-dose aspirin versus omega-3 fatty acids as adjuvants to nonsurgical periodontal therapy in Type II diabetic patients with chronic periodontitis. J Indian Soc Periodontol.

[21] Offenbacher S, Collins JG, Heasman PA (1993). Diagnostic potential of host response mediators. Adv Dent Res.

[22] Cekici A, Kantarci A, Hasturk H, Van Dyke TE (20002014). Inflammatory and immune pathways in the pathogenesis of periodontal disease. Periodontol.

[23] Raffler NA, Rivera-Nieves J, Ley K (2005). L-selectin in inflammation, infection and immunity. Drug Discov Today Ther Strateg.

[24] Hajishengallis E, Hajishengallis G (2014). Neutrophil homeostasis and periodontal health in children and adults. J Dent Res.

[25] Offenbacher S, Heasman PA, Collins JG (1993). Modulation of host PGE2 secretion as a determinant of periodontal disease expression. J Periodontol.

[26] Serhan CN (2014). Pro-resolving lipid mediators are leads for resolution physiology. Nature.

[27] Serhan CN (2007). Resolution phase of inflammation: novel endogenous anti-inflammatory and proresolving lipid mediators and pathways. Annu Rev Immunol.

[28] Mizraji G, Heyman O, Van Dyke TE, Wilensky A (2018). Resolvin D2 restrains Th1 immunity and prevents alveolar bone loss in murine periodontitis. Front Immunol.

[29] Yasuda H, Shima N, Nakagawa N (1998). Identity of osteoclastogenesis inhibitory factor (OCIF) and osteoprotegerin (OPG): a mechanism by which OPG/OCIF inhibits osteoclastogenesis in vitro. Endocrinology.

[30] Chiurchiù V, Leuti A, Dalli J, Jacobsson A, Battistini L, Maccarrone M, Serhan CN (2016). Proresolving lipid mediators resolvin D1, resolvin D2, and maresin 1 are critical in modulating T cell responses. Sci Transl Med.

[31] Ohira T, Arita M, Omori K, Recchiuti A, Van Dyke TE, Serhan CN (2010). Resolvin E1 receptor activation signals phosphorylation and phagocytosis. J Biol Chem.

[32] Arita M, Ohira T, Sun YP, Elangovan S, Chiang N, Serhan CN (2007). Resolvin E1 selectively interacts with leukotriene B4 receptor BLT1 and ChemR23 to regulate inflammation. J Immunol.

[33] Grzesik WJ, Narayanan AS (2002). Cementum and periodontal wound healing and regeneration. Crit Rev Oral Biol Med.

[34] Zhao H, Wu L, Yan G, Chen Y, Zhou M, Wu Y, Li Y (2021). Inflammation and tumor progression: signaling pathways and targeted intervention. Signal Transduct Target Ther.

[35] Bozkurt SB, Hakki SS, Kantarci A (2023). Differential effects of resolvin D1 and resolvin E1 on cementoblast function. J Periodontol.

[36] Gao L, Faibish D, Fredman G (2013). Resolvin E1 and chemokine-like receptor 1 mediate bone preservation. J Immunol.

[37] Chen J, Xu H, Xia K, Cheng S, Zhang Q (2021). Resolvin E1 accelerates pulp repair by regulating inflammation and stimulating dentin regeneration in dental pulp stem cells. Stem Cell Res Ther.

